# Spatial Image Gradient Estimation from the Diffusion MRI Profile

**DOI:** 10.1101/2025.06.06.658348

**Published:** 2025-06-10

**Authors:** Iman Aganj, Thorsten Feiweier, John E. Kirsch, Bruce R. Fischl, Andre J. van der Kouwe

**Affiliations:** 1. Athinoula A. Martinos Center for Biomedical Imaging, Radiology Department, Massachusetts General Hospital, Harvard Medical School, Boston, MA, USA; 2. Research & Clinical Translation, Magnetic Resonance, Siemens Healthineers AG, Erlangen, Germany

**Keywords:** Diffusion MRI (dMRI), spatial gradient, relaxation time, stimulated echo (STE)

## Abstract

**Background:**

In the course of diffusion, water molecules experience varying values for the relaxation-time properties of the underlying tissue, a factor that has not been accounted for in diffusion MRI (dMRI) modeling.

**Purpose:**

With the aim of mining the dMRI signal for information about the spatial variations in the tissue relaxation-time properties, we derive a new mathematical relationship between the diffusion signal and the spatial gradient of the image, which enables the estimation of the latter from the former.

**Study Type:**

Retrospective and prospective.

**Population:**

Human brain dMRI images: 617 healthy subjects from the public Human Connectome Project (HCP) Young Adults database, as well as 10 healthy volunteers and 1 *ex vivo* image scanned at our Center with stimulated-echo (STE) diffusion encoding and a long diffusion time of 1 second (which we will make publicly available).

**Field Strength/Sequence:**

3T, standard spin-echo and STE dMRI.

**Assessment:**

We validated our hypothesized relationship by evaluating the accuracy of the image spatial gradient estimated from the diffusion signal. Specifically, we compared it to the gold-standard spatial gradient approximated through finite difference, assessing the acute angle between the estimated and gold-standard gradient orientations. We furthermore measured the effects of the confounding factor of “fiber continuity”.

**Statistical Tests:**

We used two-tailed *t*-tests (α=0.05) to compare the mean/median of the abovementioned acute angle across subjects to its null-hypothesis value (57.3°/60°), hypothesizing that it would be significantly smaller.

**Results:**

We found the image gradient estimated from our diffusion model to be significantly related to that estimated via finite difference. The abovementioned acute angle had a mean/median of 51.3°/51.8° for the HCP and 53.0°/54.5° for our STE dataset, which were significantly smaller than those predicted by chance (*p* = 0 and *p* < 10^−10^, respectively). The results of fiber continuity showed an effect that was stronger than but non-overlapping with our hypothesized effect.

**Data Conclusion:**

Our results support our hypothesized relationship between within-voxel dMRI signal and image gradient, with an effect that was not explainable by the confounding factor of fiber continuity.

## 1. Introduction

As a noninvasive imaging modality, diffusion-weighted magnetic resonance imaging (dMRI) provides a wealth of information that has been proven valuable in revealing the microarchitecture of the neural tissue (1,2). Many imaging biomarkers for neurodegenerative diseases have been derived from dMRI in the past few decades (3), notably through the *in vivo* quantification of structural brain connectivity (4,5). Developing mathematical models consistent with dMRI physics and suitable to analyze dMRI data, which are inherently high-dimensional, has been necessary to extract the desired information about the tissue from the images (6–8).

Per the standard MRI model described by the Bloch equations, the MRI signal is proportional to the mean proton density (PD) inside a voxel, weighted according to relaxation times (RTs) of the tissue (9). In dMRI, where additional diffusion-encoding gradients are imposed, the signal is further attenuated with the displacement of water molecules in the direction of these gradients. Such a change in the measured signal enables inference of the diffusivity of water along different orientations, and hence the estimation of tissue properties such as fiber orientations (10,11). While the tissue RTs and diffusivity are distinct properties, they have been shown to be related to each other both theoretically (12–14) and empirically (15–19). The relationship between the two has been particularly explored in the context of diffusion-relaxation correlation (20).

Given the finite resolution of MRI, i.e. the measured signal being the average of continuous values inside a voxel, inferring within-voxel variations of an estimated quantity is often nontrivial at best. It is thus common to make approximations in MRI signal modeling by discounting such within-voxel variations. An example of such a scenario, which is the focus of this work, is in dMRI signal modeling. Although the interplay between the diffusion of spin-carrying molecules and the measured RTs has been thoroughly discussed (12–14), the effects of the spatial variations of the latter has not (to our knowledge) been accounted for in dMRI modeling. In other words, standard dMRI models assume the RT properties of the tissue (resulting in T_1_ and T_2_ weighting) to be constant inside the voxel, an assumption that disregards the (small) contribution of the within-voxel variations of these properties to the dMRI signal.

In this work, we formulate and test a hypothesis based on the premise that, in the course of diffusion, water molecules experience varying values for the RT properties of the underlying tissue. We hypothesize that the spatial variation in tissue RTs (which is related to image gradient) affects the dMRI signal, thereby creating a relationship between the diffusion profile measured by dMRI and the spatial gradient of the image. We derive this mathematical relationship and propose an approach to estimating the spatial gradient of the image from the dMRI signal (independently at each voxel). We validate our model via experiments on human brain dMRI data, specifically public images from the Human Connectome Project (HCP) Young Adult database (21), as well as stimulated-echo (STE) (22,23) images that we acquired at our Center (and will soon make publicly available; see Section 5). The STE data were acquired with a very long diffusion time of one second, which our model predicts should increase the hypothesized effect size.

We have previously presented a preliminary abstract of this work (24). In the following, we will describe our model and methods (Section 2), present experimental results (Section 3), discuss them (Section 4), and provide our code and data (Section 5).

## 2. Materials and Methods

### 2.1. Signal Modeling

The Stejskal-Tanner pulsed gradient spin-echo sequence (10) applies two gradient pulses $\vec{G}$ of duration δ, separated in time by Δ. Molecules located at ${\vec{x}}_{0}$ during the first pulse and ending up at $\vec{x}={\vec{x}}_{0}+\vec{u}$ at the second pulse presumably contribute the following to the dMRI signal $S^{v}(\vec{q})$ at voxel *v*, where $\vec{q}:=\gamma \delta \vec{G}$ is the *q*-vector (or 2*π* times it with an alternative definition) with *γ* the gyromagnetic ratio (25):

[1] $$S^{v}(\vec{q})=\underset{v}{\int }w\left({\vec{x}}_{0}\right)\rho \left({\vec{x}}_{0}\right)\text{Pr}\left(\vec{x}|{\vec{x}}_{0}\right)e^{-i\vec{q}\cdot \left(\vec{x}-{\vec{x}}_{0}\right)}\text{d}{\vec{x}}_{0}\text{d}\vec{x}\;=\underset{v}{\int }w\left({\vec{x}}_{0}\right)\rho \left({\vec{x}}_{0}\right)\text{d}{\vec{x}}_{0}\int \text{Pr}\left(\vec{u}|{\vec{x}}_{0}\right)e^{-i\vec{q}\cdot \vec{u}}\text{d}\vec{u}\;=S_{0}^{v}{\hat{P}}^{v}(\vec{q}),$$

where ρ is PD, and $w\left({\vec{x}}_{0}\right):=\left[1-\text{exp}\left(-TR/T_{1}\left({\vec{x}}_{0}\right)\right)\right]\text{exp}\left(-TE/T_{2}\left({\vec{x}}_{0}\right)\right)$ is the RT weighting for the repetition time *TR*, echo time *TE*, and the tissue longitudinal and transverse $\text{RTs},T_{1}\left({\vec{x}}_{0}\right)$ and $T_{2}\left({\vec{x}}_{0}\right)$, respectively. $S_{0}^{v}:={\langle w\rangle }_{\rho }^{v}:=\underset{v}{\int }w\left({\vec{x}}_{0}\right)\rho \left({\vec{x}}_{0}\right)\text{d}{\vec{x}}_{0}$ is the baseline non-diffusion-weighted (i.e., b=0) image, where ${\langle \cdot \rangle }_{\rho }^{v}$ denotes *ρ*-weighted sum inside *v*, i.e. the voxel. ${\hat{P}}^{v}:=ℱ\left\{P^{v}\right\}$ is the Fourier transform of $P^{v}(\vec{u}):=\text{Pr}(\vec{u})≅\text{Pr}\left(\vec{u}|{\vec{x}}_{0}\right)$, which is the probability of diffusion with the amount $\vec{u}$ (a.k.a. ensemble average propagator) during the effective diffusion time τ:=Δ−δ/3, and is presumed (25) independent of ${\vec{x}}_{0}\in v$ within the voxel (although it could be considered dependent on the tissue type in multicompartment models).

The RT properties of tissue have been assumed to be constant along the trajectory of the diffusing water, thereby simplifying the above model. Given that the spatial distribution of molecules diffusing from ${\vec{x}}_{0}$ to $\vec{x}$ is their *initial* density, the integrals in Eq. [1] are weighted by $\rho \left({\vec{x}}_{0}\right)$. However, *w* is expected to vary in the tissue continuum along the molecule’s trajectory, which would affect the resulting signal attenuation. To account for the variations of *w*, the integral needs to be weighted by an *effective* value of *w* experienced by the molecules going from ${\vec{x}}_{0}$ to $\vec{x}$, rather than by its initial value at ${\vec{x}}_{0}$. Thus, weighting the above integral by $w\left({\vec{x}}_{0}\right)$, as done in the state-of-the-art dMRI models, neglects how within-voxel variation of *w* affects the dMRI signal. Such effects could in fact be exploited to further learn about the tissue microstructure.

We propose to use an effective value for the RT weighting, *w*, to account for its change during a molecule’s diffusion. For particles going from ${\vec{x}}_{0}$ to $\vec{x}$, instead of weighting the integral in Eq. [1] by the initial value $w\left({\vec{x}}_{0}\right)$, we will use the *midpoint* value,

[2] $$w\left(½\left({\vec{x}}_{0}+\vec{x}\right)\right)=w\left({\vec{x}}_{0}+½\vec{u}\right)\;≅w\left({\vec{x}}_{0}\right)+½\nabla_{x}w\left({\vec{x}}_{0}\right)\cdot \vec{u},$$

where $\nabla_{x}$ is the spatial gradient. This leads to:

[3] $$S^{v}(\vec{q})=\underset{v}{\int }w\left({\vec{x}}_{0}\right)\rho \left({\vec{x}}_{0}\right)\text{d}{\vec{x}}_{0}\int P^{v}(\vec{u})e^{-i\vec{q}\cdot \vec{u}}\text{d}\vec{u}+½\underset{v}{\int }\nabla_{x}w\left({\vec{x}}_{0}\right)\rho \left({\vec{x}}_{0}\right)\text{d}{\vec{x}}_{0}\cdot \int \vec{u}P^{v}(\vec{u})e^{-i\vec{q}\cdot \vec{u}}\text{d}\vec{u}\;={\langle w\rangle }_{\rho }^{v}ℱ\left\{P^{v}(\vec{u})\right\}+½{\langle \nabla_{x}w\rangle }_{\rho }^{v}\cdot ℱ\left\{\vec{u}P^{v}(\vec{u})\right\}\;=S_{0}^{v}{\hat{P}}^{v}(\vec{q})+½i{\langle \nabla_{x}w\rangle }_{\rho }^{v}\cdot \nabla_{q}{\hat{P}}^{v}(\vec{q}),$$

where we used the relationship $ℱ\left\{\vec{u}P^{v}(\vec{u})\right\}=i\nabla_{q}{\hat{P}}^{v}(\vec{q})$, with $\nabla_{q}$ the gradient with respect to $\vec{q}.$ Note that our linear approximation of *w* in Eq. [2] is local only within the molecule’s trajectory and does not extend to the entire voxel.

Provided the measurements of the diffusion signal $S^{v}(\vec{q})$ for many *q*-vectors, as is common in dMRI, Eq. [3] allows the estimation of ${\langle \nabla_{x}w\rangle }_{\rho }^{v}$, i.e. the mean $\nabla_{x}w$ (weighted by PD) within the voxel, which can reveal potentially new information about the tissue microarchitecture. By contributing to the imaginary part of the signal, $\nabla_{x}w$ affects both the magnitude and the phase of the signal. It has been shown that the background phase varies strongly from one diffusion image to another in the scan (due to physiological factors) (26,27), which would render the estimation of the contribution of ${\langle \nabla_{x}w\rangle }_{\rho }^{v}$ to the phase image impractical. The *magnitude* of the signal, which in contrast remains largely consistent from shot to shot, would be:

[4] $$\left|S^{v}(\vec{q})\right|=S_{0}^{v}{\hat{P}}^{v}(\vec{q})\sqrt{1+{\left({\vec{L}}^{v}\cdot ½\nabla_{q}\text{log}{\hat{P}}^{v}(\vec{q})\right)}^{2}},$$

where ${\vec{L}}^{v}:={\langle \nabla_{x}w\rangle }_{\rho }^{v}/S_{0}^{v}$, and we made the standard assumption that diffusion is symmetric (thus ${\hat{P}}^{v}(\vec{q})$ is real), as well as used $\nabla_{q}{\hat{P}}^{v}(\vec{q})/{\hat{P}}^{v}(\vec{q})=\nabla_{q}\text{log}{\hat{P}}^{v}(\vec{q})$. In the simple case of Gaussian diffusion, the diffusion tensor imaging (DTI) model (11) predicts ${\hat{P}}^{v}(\vec{q})=\text{exp}\left(-\tau {\vec{q}}^{T}D^{v}\vec{q}\right)$, where $D^{v}$ is the symmetric diffusion tensor at voxel *v*, resulting in the factor $½\nabla_{q}\text{log}{\hat{P}}^{v}(\vec{q})=-\tau D^{v}\vec{q}$ being a linear function of $\vec{q}$. The DTI approximation, therefore, leads to:

[5] $$\left|S^{v}(\vec{q})\right|≅S_{0}^{v}e^{-\tau {\vec{q}}^{T}D^{v}\vec{q}}\sqrt{1+{\left(\tau {\vec{L}}^{v^{T}}D^{v}\vec{q}\right)}^{2}}.$$

With this model, measurements of $\left|S^{v}(\vec{q})\right|$ for at least 9 values of $\vec{q}$ in different orientations (in addition to an $S_{0}^{v}$ image) would be required to estimate the 6 parameters of $D^{v}$ and the 3 elements of the vector ${\vec{L}}^{v}$ (and subsequently ${\langle \nabla_{x}w\rangle }_{\rho }^{v}$. Note that, since $\vec{L^{v}}$ is raised to the power of 2 in Eq. [5], its sign cannot be directly recovered from $\left|S^{v}(\vec{q})\right|$ measurements, meaning that its magnitude and *orientation* (rather than direction) can be estimated.

### 2.2. Implementation and Assessment

To estimate $D^{v}$ and ${\vec{L}}^{v}$ at each voxel *v*, we fit the values of the diffusion signal for all available $\vec{q}$ to Eq. [5] via the pattern search algorithm (28), while initializing $D^{v}$ with standard DTI reconstruction (11) and ${\vec{L}}^{v}$ as a 3×1 vector of all-zeros.

We assess the orientational accuracy of the estimated ${\vec{L}}^{v}$ by comparing it to its discrete counterpart (used as the gold standard) computed via finite difference, testing the hypothesis that the two orientations are more aligned than expected by chance. Where the PD image, ${\langle 1\rangle }_{\rho }^{\nu }$, is available, the discrete counterpart of ${\langle \nabla_{x}w\rangle }_{\rho }^{v}$ would be ${\langle 1\rangle }_{\rho }^{v}\partial_{\text{fd}}\left({\langle w\rangle }_{\rho }^{v}/{\langle 1\rangle }_{\rho }^{v}\right)={\langle 1\rangle }_{\rho }^{v}\partial_{\text{fd}}\left(S_{0}^{v}/{\langle 1\rangle }_{\rho }^{v}\right)$, with $\partial_{\text{fd}}$ denoting the finite-difference gradient operator. The discrete counterpart of ${\vec{L}}^{v}$ can therefore be computed as the following vector:

[6] $$\partial_{\text{fd}}\left(\frac{S_{0}^{v}}{{\langle 1\rangle }_{\rho }^{v}}\right)/\left(\frac{S_{0}^{v}}{{\langle 1\rangle }_{\rho }^{v}}\right)\;≅\partial_{\text{fd}}\text{log}\left(\frac{S_{0}^{v}}{{\langle 1\rangle }_{\rho }^{v}}\right)\;=\partial_{\text{fd}}\text{log}\left(S_{0}^{v}\right)-\partial_{\text{fd}}\text{log}\left({\langle 1\rangle }_{\rho }^{v}\right).$$

However, since the PD image is often unavailable in dMRI datasets, we approximate the above discrete counterpart with its first term, $\partial_{\text{fd}}\text{log}\left(S_{0}^{v}\right)$. Given that the $S_{0}$ and PD $\left({\langle 1\rangle }_{\rho }^{v}\right)$ image intensities both correlate with the same underlying tissue, their gradient *orientations* are expected to align with each other, favoring our approximation.

We measure the acute angle (0 ≤ *θ* ≤ 90°) between the orientations of the estimated ${\vec{L}}^{v}$ and its discrete counterpart, which should be small if the two are similarly oriented. The null hypothesis, i.e. ${\vec{L}}^{v}$ is randomly oriented with respect to its discrete counterpart, predicts the null probability density distribution of *θ* to be sin *θ* (due to the infinitesimal solid angle dΩ=sinθdθdϕ, with the mean of 57.3° (1 rad) and the median of 60°. We use two-sided *t*-tests to see if the population-averages of the mean and median of *θ* are different from these null values.

### 2.3. Effect Size

The diffusivity of the tissue can be quantified from $-\text{log}\left(\left|S^{v}(\vec{q})\right|/S_{0}^{v}\right)$. In our case, using the DTI model, that would be:

[7] $$-\text{log}\frac{\left|S^{v}(\vec{q})\right|}{S_{0}^{v}}=\tau {\vec{q}}^{T}D^{v}\vec{q}-\text{log}\sqrt{1+{\left(\tau {\vec{L}}^{v^{T}}D^{v}\vec{q}\right)}^{2}}\;≅\tau {\vec{q}}^{T}D^{v}\vec{q}-½{\left(\tau {\vec{L}}^{v^{T}}D^{v}\vec{q}\right)}^{2}\;=\tau {\vec{q}}^{T}D^{v}\left(I-½\tau {\vec{L}}^{v}{\vec{L}}^{v^{T}}D^{v}\right)\vec{q}.$$

The relative contribution of ${\vec{L}}^{v}$ to the above quantity, which we call the dimensionless *effect size*, is of the magnitude $½\tau {\left\|{\vec{L}}^{v}\right\|}_{2}^{2}\left\|D^{v}\right\|$. With the simplifying assumptions that PD remains relatively constant inside the voxel and *w* varies with a general linear trend, one can see ${\left\|{\vec{L}}^{v}\right\|}_{2}^{2}$ to be largely bounded by $4\left(1/s_{x}^{2}+1/s_{y}^{2}+1/s_{z}^{2}\right)$, where s_a_ is the pixel size in the *a*-axis (these assumptions are used only here to derive this closed-form upper bound, and nowhere else). Therefore, for a dMRI of the brain white matter (WM) with $s_{x}=s_{y}=s_{z}=1.25\text{mm},\tau =40\text{ms}$, and $\left\|D^{v}\right\|=0.0007{\text{mm}}^{2}/\text{s}$, the effect size would be about 10^−4^ (enabling the approximation in Eq. [7]), which might be too small to detect. The effect size can however be increased using a long *τ*. This prompted us to test our hypothesis additionally with the STE sequence (22,23) that can achieve a diffusion time of *τ* = 1 s (Section 2.4.2), while preserving the signal-to-noise ratio (SNR) by not increasing the effective TE (that would otherwise cause T_2_ signal loss).

### 2.4. Population

We tested our algorithm on the following two datasets.

### 2.4.1. Human Connectome Project (HCP) – Young Adult

We processed dMRI data of 617 subjects from the public WashU-UMN HCP Young Adult (21). The images had been acquired on a customized Siemens 3T scanner at the 1.25 mm isotropic resolution, with the diffusion time of *τ_HCP_* = 40 ms, along 270 diffusion gradient directions with b-values ranging from 990 to 3010 s/mm^2^, in addition to 18 b=0 s/mm^2^ (*S*_0_) images. We used the brain (gray and white matter) mask computed from the T_1_ image via FreeSurfer (29), resampled it in the dMRI space, and refined it as described in Section 2.5.

### 2.4.2. Acquired Stimulated-Echo (STE) dMRI

We used an STE dMRI (22,23) research sequence with the long diffusion time of *τ* = 1 s to acquire the following images on a 3T scanner (MAGNETOM Skyra, Siemens Healthineers AG, Forchheim, Germany) with a 32-channel head coil, GRAPPA acceleration 2, b-value of 1000 s/mm^2^, and isotropic voxel size of 2 mm. A rough mask was used for analysis, as described in Section 2.5.

We scanned 10 healthy volunteers (7 females, age: 36 ± 14 years old), whose consent and data were collected as part of an existing project to develop novel MRI acquisition software, approved by the Institutional Review Board of Mass General Brigham. We used the receiver bandwidth 1658 Hz/px, TR of 26400 ms, effective TE of 31 ms, and image size of 104×104×25 voxels. We acquired images with 64 diffusion directions, as well as 20 repetitions of the b=0 image, resulting in a total acquisition time of 37.5 minutes.

In addition, we scanned an *ex vivo* sample of a brain hemisphere using the TR of 26200 ms, effective TE of 33 ms, receiver bandwidth 1698 Hz/px, and image size of 128×128×25 voxels. To increase the SNR, we acquired images with 256 diffusion directions, with 8 repetitions per direction, as well as 320 repetitions of the b=0 image, resulting in a total acquisition time of 17.3 hours. The repeated images were averaged before further analysis.

See Section 5 for the public availability of this long-diffusion-time STE dMRI dataset.

### 2.5. Fiber Continuity: A Confounding Factor

Stemming from dMRI physics, our hypothesis is a relationship between diffusional information in the signal and spatial derivative of the image. However, characteristics of the fibrous tissue can additionally cause the fiber orientation to be confoundingly related to edges in the image. In particular, due to fiber continuity (30–32), fiber bundles vary smoothly along their own orientations, meaning that image edges would likely not be perpendicular to orientations with high diffusion. This means that the spatial gradient of the image in the fiber bundles is likely stronger along the lower- rather than higher-diffusion orientations.

In designing our experiments, we attempted to heuristically avoid the areas affected by fiber continuity by limiting our analysis to WM regions that are far from the bundle edges. For our *in vivo* (HCP and STE) images, we only kept voxels with fractional anisotropy (FA) greater than 0.4, b=0 image intensity greater than 80, and mean apparent diffusion coefficient (ADC) greater than 0.0002 mm^2^/s and smaller than 0.0008 mm^2^/s (HCP) or 0.0009 mm^2^/s (STE). For our *ex vivo* STE image, we used a rough mask including only regions with FA greater than 0.25, mean ADC smaller than 0.0003 mm^2^/s, and b=0 image intensity greater than 90.

Furthermore, for comparison, in addition to estimating the orientation of the spatial gradient of the image using our hypothesized relationship (${\vec{L}}^{v}$ in Eq. [5]), we also estimated it following the fiber continuity assumption as the orientation of the eigenvector corresponding to the smallest eigenvalue of the diffusion tensor, calling it ${\vec{F}}^{v}$.

## 3. Results

### 3.1.1. HCP Results

**Figure 1.**
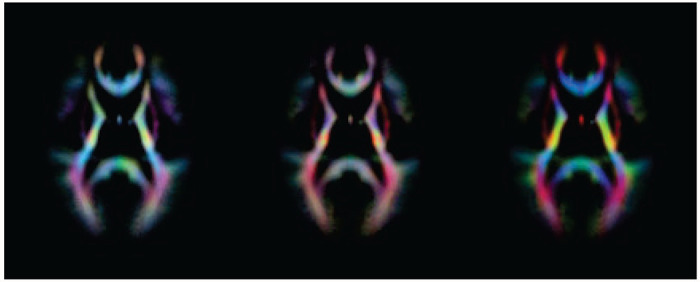
Axial view of the spatial gradient of the image estimated from the diffusion profile using the proposed (left) and fiber-continuity (right) approaches, and from the b=0 image using the finite-difference approach (middle). Colors show the strength of the gradient at each spatial coordinate after normalizing the gradient, taking its absolute value, and averaging across all HCP subjects (not to be confused with color-coded FA).

We estimated the proposed ${\vec{L}}^{v}$, the fiber-continuity-derived ${\vec{F}}^{v}$, and their gold-standard discrete counterpart for each subject. Figure 1 shows the average of each of the three (normalized) vector fields across all the 617 subjects, color-coded with respect to the estimated orientation. One can see that the spatial-gradient orientations derived from both our (left) and fiber-continuity (right) approaches visually correspond to some extent to the discretely computed orientations (middle).

**Figure 2.**
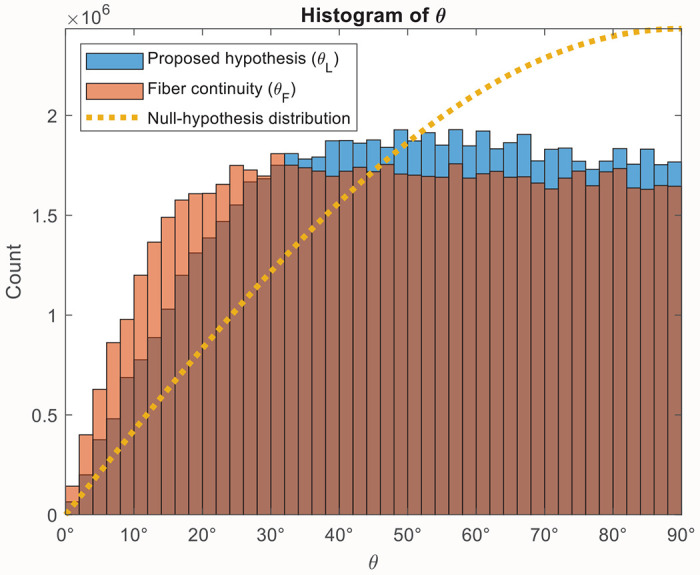
Histograms of the acute angles between spatial gradient of the image computed discretely from the b=0 image and the ones estimated from the diffusion profile using the proposed (*θ_L_*, blue) and the fiber-continuity (*θ_F_*, red) approaches, across all WM voxels of all HCP subjects. The dotted orange line is the distribution of this angle under the null hypothesis (i.e., if the two orientations were not related).

The histogram of the acute angle *θ_L_* between the estimated ${\vec{L}}^{v}$ and its discrete gold-standard counterpart across all WM voxels of all subjects is plotted in Figure 2. The distribution of *θ_L_* (blue), which is visibly shifted to the left compared to the null hypothesis (dashed curve), has a mean/median of 51.3°/51.8°, i.e. smaller than chance (57.3°/60°). A similar histogram of *θ_F_* between the fiber-continuity-derived ${\vec{F}}^{v}$ and the discrete counterpart is shown in red, which is further to the left, with its mean and median both being 48.6°.

**Figure 3.**
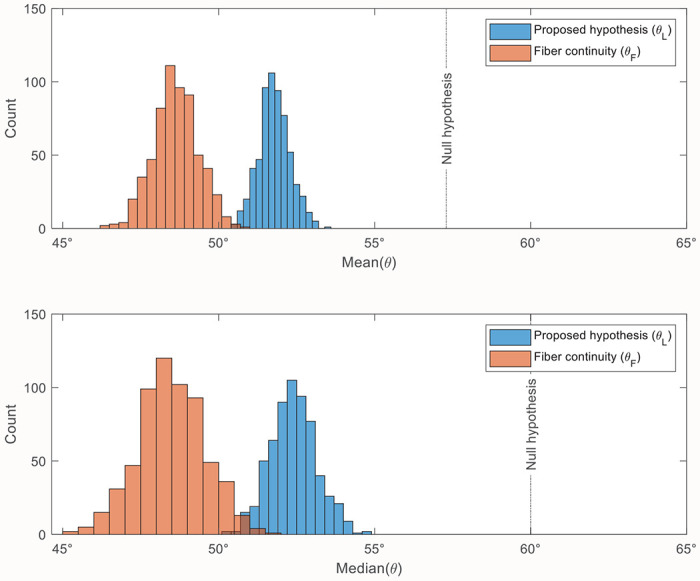
Histograms of the within-subject mean (top) and median (bottom) of the acute angles between spatial gradient of the image computed discretely from the b=0 image and the ones estimated from the diffusion profile using the proposed (*θ_L_*, blue) and the fiber-continuity (*θ_F_*, red) approaches, across all HCP subjects. The dashed vertical lines indicate the values under the null hypothesis (i.e., if the two orientations were not related).

Next, we computed the mean/median of both *θ_L_* and *θ_F_* for each subject separately, the histograms of which across subjects are plotted in Figure 3. Two-tailed *t*-tests revealed these values to be significantly smaller than those predicted by chance (*p* = 0, within the double-precision limits).

**Figure 4.**
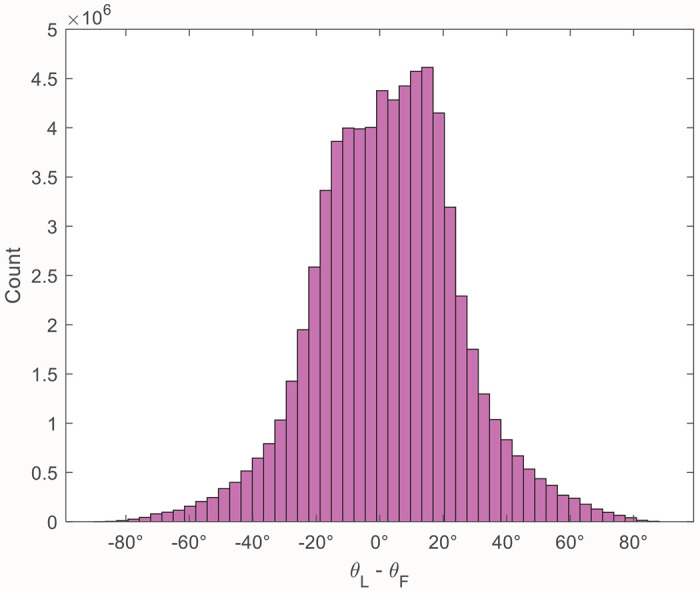
Histogram of *Θ_L_ – Θ_F_* across all WM voxels of all HCP subjects. 45% of the voxels are on the negative side (*Θ_L_ < Θ_F_*) of the distribution.

We then compared *θ_L_* and *θ_F_* voxel-wise. Even though *θ_L_* was on average slightly larger than *θ_F_* (see above), the histogram of *θ_L_ – θ_F_*, plotted in Figure 4, revealed a considerable portion (45%) of voxels with *θ_L_ < θ_F_*, implying *non-overlapping* orientational information mined by the two approaches.

#### 3.1.2. STE Results

**Figure 5.**
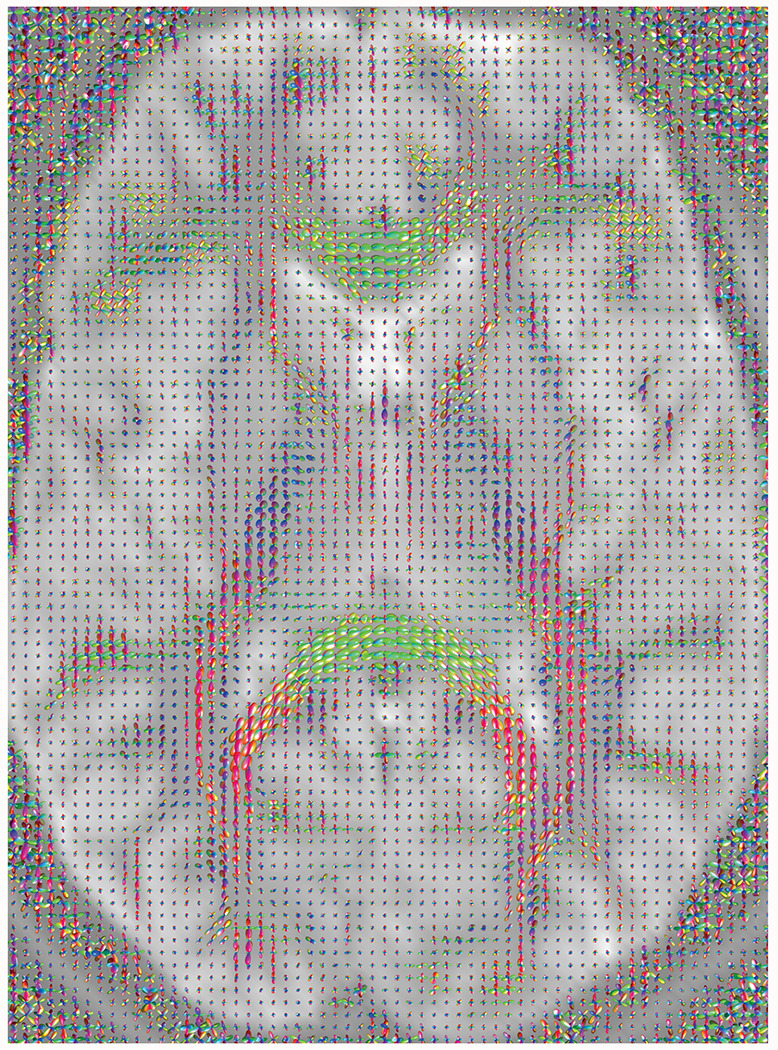
Axial view of the q-ball CSA-ODFs reconstructed from the STE data of a representative participant.

Figure 5 shows the q-ball orientation distribution functions in constant solid angle (CSA-ODFs) (33) reconstructed with a (real and symmetric) spherical harmonic order of 4 and visualized using our public MATLAB (The MathWorks Inc., Natick, MA, USA) toolbox (34) (see Section 5) for a representative subject in our *in vivo* STE dataset.

An analysis similar to Section 3.1.1 for our *in vivo* STE images revealed the mean/median of *θ_L_* and *θ_F_* to be 53.0°/54.5° and 44.7°/42.7°, respectively, which are again smaller than chance (57.3°/60°). Within-subject mean/median of both *θ_L_* and *θ_F_* were significantly smaller than those predicted by chance (cross-subject *t*-test *p* < 10^−10^). Comparing *θ_L_* and *θ_F_* voxel-wise revealed 39% of voxels with *θ_L_ < θ_L_*.

**Figure 6.**
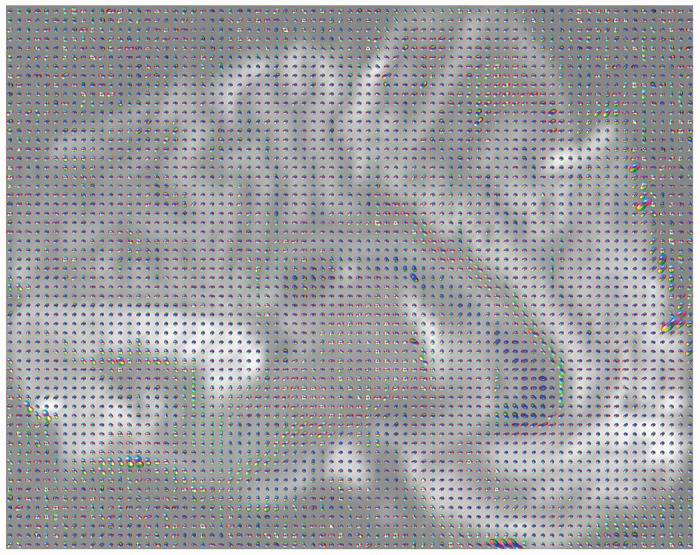
Sagittal view of the DTI CSA-ODFs reconstructed from the *ex vivo* STE scan.

Regarding our *ex vivo* STE image, the CSA-ODFs reconstructed from the diffusion tensors are depicted in Figure 6. The mean/median of *θ_L_* and *θ_F_* for this image were 56.5°/58.7° and 53.5°/55.2°, respectively, which are smaller than chance (but not by as much as the *in vivo* results), with 46% of voxels showing *θ_L_ < θ_L_*.

## 4. Discussion

We have introduced a model that takes advantage of diffusional information in the dMRI signal to infer new spatial information, thereby deriving a new relationship between the diffusion profile and the spatial gradient of tissue RT weighting. We evaluated our model on 617 brain dMRI images from the public HCP database, as well as on 10 *in vivo* and 1 *ex vivo* brain STE images that we acquired at our Center. We observed the effect of our hypothesized relationship in all our results; namely, the spatial-gradient orientation estimated from the diffusion profile using our relationship was statistically significantly closer to the gold-standard orientation (approximated through finite difference) than predicted by chance. While the relationship between diffusional and RT properties of the tissue has been discussed (12–14) and measured (15–20,35) in the literature, our proposed framework is – to the best of our knowledge – the first to enable the estimation of the within-voxel spatial gradient of the RT weighting from the dMRI signal.

The decision to test our hypothesis additionally on the STE images was motivated by the theoretical prediction that the spatial image gradient affects the signal more strongly at longer diffusion times (Section 2.3). Our STE protocol with *τ* = 1 s indeed provided us with a diffusion time 25 times longer than that of HCP (*τ_HCP_* = 40 ms). Nevertheless, the effect observed in the STE images was not as strong as that in the standard dMRI images of HCP, possibly due to other differences between the two datasets (see Section 4.1).

A confounding factor in validating our hypothesis was the concept of fiber continuity (30–32), a characteristic of the fibrous tissue that implies smooth variation of a fiber bundle along its orientation, hence smaller image gradient along high-diffusion orientations (see Section 2.5). To distinguish the relationship derived from our dMRI-physics hypothesis from that due to fiber continuity, we also estimated the spatial gradient of the image from the latter relationship, which produced, on average, more accurate spatial-gradient orientations (when compared to the gold standard) than our hypothesis did. Nevertheless, we observed that in a substantial portion of the voxels (45% in HCP dMRI, 39% in *in vivo* STE, and 46% in *ex vivo* STE), our hypothesized relationship still led to more accurate orientation estimation than fiber continuity did. This implies that, regardless of the fiber continuity effect being stronger than our hypothesized effect, the latter is not simply a weaker version of the former. In other words, our observations rule out the (full) overlap of the two effects, which can be clearly observed in the broad distribution of the difference between the acute angles estimated from our and fiber-continuity approaches (Figure 4).

Potential future directions would be to investigate whether the spatial information hidden within the diffusion profile could: 1) help to improve the characterization of fine WM pathways and cortical microstructure, 2) be used as a dMRI biomarker for disease, and 3) be exploited as supplemental input to improve dMRI super-resolution algorithms (36–39). The latter is especially important as higher dMRI spatial resolution reduces the unwanted mixture of tissues in a voxel (partial volume effect), enhancing the precision and reliability of diffusion modeling and tractography (7,40–42).

Our STE dMRI dataset, which is publicly available (see Section 5), is unique in that it has been acquired with an unusually long diffusion time of *τ* = 1 s. This dataset includes *in vivo* brain images of 10 healthy volunteers and 1 *ex vivo* brain image. While we collected these data specifically to test the hypothesis proposed here, we hope that this dataset will be useful for the research community to study the diffusion properties of the neural tissue at very long diffusion times (43), e.g. the restricted diffusion (44), and test other hypotheses. Public STE datasets by other groups, e.g. of the human heart (45) or the mouse spinal cord (46), can further facilitate such efforts.

### 4.1. Limitations

Although we showed that the effects of the proposed model did not fully overlap with those of fiber continuity, our significant results may still be due to a mix of the two effects. Further evaluation, e.g. on physical phantoms constructed while minimizing the fiber continuity effect, can be helpful to conclusively validate our hypothesis.

In our evaluation, where the discretely computed image gradient was used for comparison, we made an approximation by ignoring the variations in the PD image in Eq. [6]. Nonetheless, since we used only the orientation (but not the magnitude) of the spatial gradient for validation, and given that the gradients of the PD and b=0 images are both related to the same underlying tissue and thus oriented similarly to each other, the aforementioned approximation is not expected to have substantially affected the accuracy of our validation. Another consideration about our evaluation is that the gold-standard gradient was computed as the *central* finite difference, which, by using multiple voxels, introduces some blurring that is not expected in our diffusion-based model, hence a discrepancy in the comparison.

Our hypothesized effect was observed to be weaker for the STE compared to the HCP data, possibly because of the larger voxels of the STE images (*s_x_ = s_y_ = s_z_* = 2 mm, compared to *s_x_ = s_y_ = s_z_* = 1.25 mm of the HCP; see Section 2.3), lower SNR (two-time reduction in signal strength) inherent to STE (23), inadequacy of the DTI (Gaussian) model at long *τ* (47), and less specific WM mask.

### 4.2. Conclusion

We extended the standard dMRI reconstruction model to account for within-voxel variation of the RT properties of the tissue, and derived a closed-form relationship in the case of DTI. The new mathematical model enables the estimation of the spatial image gradient from the diffusion profile. We evaluated our model on public HCP data, as well as a unique in-house STE dataset that we acquired with a very long diffusion time (and provide freely to the public). Our experimental results support the validity of our hypothesis with statistical significance. Future work consists of leveraging the estimated spatial gradient to learn about tissue microstructure, discover biomarkers, and increase the spatial resolution of dMRI.

## 5. Data and Code Availability

We are currently curating and anonymizing the STE data described in Section 2.4.2. We will provide the de-identified dataset to the public soon, thereby including the link to the data repository in this section before acceptance of this manuscript.

Diffusion and STE fiber orientation reconstruction was performed using our public CSA-ODF and Hough-Tractography toolbox (34) (www.nitrc.org/projects/csaodf-hough). We are currently making our code for the proposed spatial-gradient estimation user-friendly and will incorporate it in the same publicly available toolbox before acceptance of this manuscript.

The public WashU-UMN Human Connectome Project (HCP) Young Adult (21) database is available at: https://www.humanconnectome.org/study/hcp-young-adult/data-releases
